# Co-targeting of DNA, RNA, and protein molecules provides optimal outcomes for treating osteosarcoma and pulmonary metastasis in spontaneous and experimental metastasis mouse models

**DOI:** 10.18632/oncotarget.16372

**Published:** 2017-03-18

**Authors:** Chao Jian, Mei-Juan Tu, Pui Yan Ho, Zhijian Duan, Qianyu Zhang, Jing-Xin Qiu, Ralph W. DeVere White, Theodore Wun, Primo N. Lara, Kit S. Lam, Ai-Xi Yu, Ai-Ming Yu

**Affiliations:** ^1^ Department of Orthopedics, Zhongnan Hospital of Wuhan University, Wuhan, Hubei, China; ^2^ Department of Biochemistry & Molecular Medicine, UC Davis School of Medicine, Sacramento, CA, USA; ^3^ Department of Pathology, Roswell Park Cancer Institute, Buffalo, NY, USA; ^4^ Department of Urology, UC Davis School of Medicine, Sacramento, CA, USA; ^5^ Division of Hematology Oncology, UC Davis School of Medicine, Sacramento, CA, USA; ^6^ Department of Internal Medicine, Comprehensive Cancer Center, UC Davis School of Medicine, Sacramento, CA, USA

**Keywords:** therapy, metastasis, doxorubicin, miR-34a, sorafenib, Pathology Section

## Abstract

Metastasis is a major cause of mortality for cancer patients and remains as the greatest challenge in cancer therapy. Driven by multiple factors, metastasis may not be controlled by the inhibition of single target. This study was aimed at assessing the hypothesis that drugs could be rationally combined to co-target critical DNA, RNA and protein molecules to achieve saturation attack against metastasis. Independent actions of the model drugs DNA-intercalating doxorubicin, RNA-interfering miR-34a and protein-inhibiting sorafenib on DNA replication, RNA translation and protein kinase signaling in highly metastatic, human osteosarcoma 143B cells were demonstrated by the increase of? H2A.X foci formation, reduction of c-MET expression and inhibition of Erk1/2 phosphorylation, respectively, and optimal effects were found for triple-drug combination. Consequently, triple-drug treatment showed a strong synergism in suppressing 143B cell proliferation and the greatest effects in reducing cell invasion. Compared to single- and dual-drug treatment, triple-drug therapy suppressed pulmonary metastases and orthotopic osteosarcoma progression to significantly greater degrees in orthotopic osteosarcoma xenograft/spontaneous metastases mouse models, while none showed significant toxicity. In addition, triple-drug therapy improved the overall survival to the greatest extent in experimental metastases mouse models. These findings demonstrate co-targeting of DNA, RNA and protein molecules as a novel therapeutic strategy for the treatment of metastasis.

## INTRODUCTION

Metastasis remains a major cause of death of cancer patients. Approximately 90% of cancer-associated mortality arises from systemic metastasis of the primary tumor [1–3]. As an example, osteosarcoma (OS), a primary locally destructive mesenchymal tumor accounting for 60% of malignant bone tumors among children and young adults, has a predilection for lung metastases [4–6]. Improved therapies for OS have increased the 5-year survival rate to 60-70% for non-metastatic OS of the extremities [7–9], dramatically extending the life span of childhood OS survivors. However, the 5-years survival rate is only 18-33% for those with lung metastases at the time of diagnosis [9–11]. Overall 80% of OS patients die from distant metastases, most commonly in the lungs [9].

The processes such as invasion from the primary site, circulation of tumor-initiating cells, metastatic colonization, and distant outgrowth of metastases underlying cancer invasion-metastasis cascade are very complex [1–3]. While some molecular determinants and signaling pathways (e.g., c-MET, VEGF, Ras-Raf-MAPK-ERK pathways, etc.) behind cancer metastatic processes have been identified, much remains poorly understood. Furthermore, the presence of redundant signaling pathways and the emergence of complementary mechanisms and resistant phenotypes limit the efficacy of targeted anti-neoplastic therapies. Therefore, combating metastasis remains one of the greatest challenges in cancer therapy [3, 12] and blocking single target or pathway is unlikely to be very effective.

We recently established a novel platform for high-yield production of biologic RNA-interfering (RNAi) agents including miRNAs and siRNAs [13, 14] that are distinguished from commonly used synthetic RNAi reagents carrying extensive artificial modifications [15]. We also demonstrated that biologic miR-34a prodrug was precisely processed to mature miR-34a and thus effective to regulate target gene expression (e.g., c-MET) [14, 16]. In addition, combination therapy with miR-34a prodrug and doxorubicin exerted synergistic effects in repressing human OS cell growth *via* RNA interference and DNA intercalation, and this combination is more effective than monotherapy in suppressing OS xenograft tumor growth [17].

**Figure 1:**
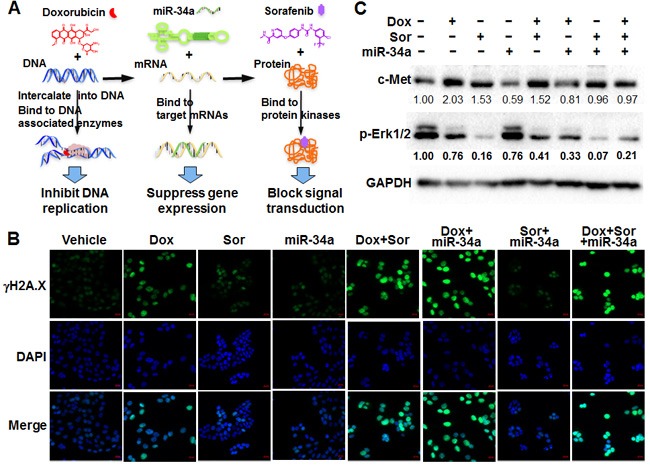
Independent and combined effects of triple drugs on DNA, mRNA and protein targets in highly metastatic osteosarcoma cells **A**. Doxorubicin (Dox), bioengineered miR-34a prodrug (miR-34a) and sorafenib (Sor) were chosen as model drugs to target DNA and particular mRNAs and proteins, respectively. **B**. Immunofluorescent analysis of γH2A.X foci formation indicated Dox-induced DNA damage in 143B cells, which were enhanced to greater degrees with the addition of miR-34a or Sor. Cells were fixed at 24 h post-treatment and stained with specific γH2A.X antibody and then corresponding Alexa Fluor 488-conjugated antibody. Images were acquired with a confocal microscope. The bar indicates 20 μm. **C**. Immunoblot analysis of protein levels of p-Erk1/2 and c-Met confirmed the actions of Sor and miR-34a, respectively. Band density was determined by Image Lab software (Bio-Rad), and normalized to that of GAPDH. The protein levels in vehicle group were defined as 1.00.

To explore new strategy for the treatment of metastasis, we hypothesized that optimal outcomes might be achieved through “saturation attack”, i.e., co-targeting of DNA, and particular RNA and protein molecules (Figure 1A) critical for almost all cancer cellular processes. To test the hypothesis, we employed DNA-intercalating doxorubicin, RNA-interfering miR-34a, and protein kinase-inhibiting sorafenib as model drugs and assessed their independent and complementary actions in highly metastatic, human OS 143B model cells. Doxorubicin is one of the chemotherapeutic agents (i.e., methotrexate, doxorubicin, and cisplatin; sometimes with ifosfamide) that are currently used for the treatment of soft tissue sarcomas [7]. Since VEGFR and Raf/MEK/ERK signaling pathways have been reported to be relevant to OS progression [18–20], sorafenib was chosen as a protein kinase-inhibiting model drug. Because miR-34a is significantly downregulated in human OS tissues [18, 19] and reintroduction of miR-34a into OS cells inhibits cell proliferation and suppresses tumorigenesis [20, 21], miR-34a was selected as an RNA-interfering agent. In addition, the biologic miR-34a prodrug used in this study was produced and folded in live cells [13, 14], which is distinguished from synthetic miRNA agents bearing extensive artificial modifications [15, 22]. As a result, the combined drugs showed a strong synergism and the greatest effects in suppressing OS cell proliferation and invasion. In spontaneous and experimental metastasis mouse models, rational triple-drug combination therapy elicited the most beneficial outcomes, as manifested by the highest degrees of suppression of orthotopic OS progression and pulmonary metastases, as well as improvement of survival.

## RESULTS

### Independent and combined actions of doxorubicin, sorafenib and miR-34a on target molecules in metastatic 143B cells

To test the concept of co-targeting of critical DNA, RNA and protein molecules in metastatic cancer cells (Figure 1A), we first examined the effects of model drugs doxorubicin, miR-34a and sorafenib on corresponding targets and marker proteins in OS 143B cells and defined their interactions when used in combination. An immunofluorescent study was carried out to investigate γH2A.X foci formation, which is a sensitive and robust biomarker of DNA damage [23] induced by DNA-intercalating drugs and may indicate the mechanistic actions of doxorubicin. Compared to 143B cells treated with vehicle, sorafenib plus miR-34a treatment did not alter γH2A.X signal intensity while doxorubicin alone sharply induced the damage of DNA (Figure 1B). Moreover, co-administration of sorafenib and miR-34a, alone or together, with doxorubicin sharply increased the levels of γH2A.X. The highest levels were observed in cells treated with doxorubicin plus miR-34a, with or without sorafenib (Figure 1B). The same results were obtained by Western blot analyses of γH2A.X (data not shown), indicating that sorafenib or miR-34a alone or combined does not alter γH2A.X foci formation but is able to largely enhance doxorubicin-provoked DNA damage in 143B cells.

Western blots were performed to verify the effects of miR-34a and sorafenib as well as combinatorial effects in 143B cells (Figure 1C). Compared to vehicle treatment, miR-34a led to a 40% suppression of c-MET protein levels, a well-defined miR-34a target [24–26] being involved in osteosarcoma metastasis [27–29]. To our surprise, either doxorubicin or sorafenib alone or in combination led to an upregulation of c-MET. However, co-administration of miR-34a abrogated the increase of c-MET expression caused by doxorubicin and sorafenib (Figure 1C). Likewise, the inhibitory effects of sorafenib on protein kinases [30–32] were indicated by an 80% reduction in phosphorylation of target Erk1/2, as revealed by Western blot analyses of p-Erk1/2 (Figure 1C). While doxorubicin and miR-34a alone caused ~20% inhibition of p-Erk1/2 levels, triple-drug combination reduced p-Erk1/2 to a comparable level as sorafenib alone, which might had reached a saturation point. Together, the results confirmed the independent actions of doxorubicin, sorafenib and miR-34a in 143B cells as well as the ultimate combinatorial effects.

### Synergism of doxorubicin, sorafenib and miR-34a in the suppression of 143B cell proliferation

**Figure 2:**
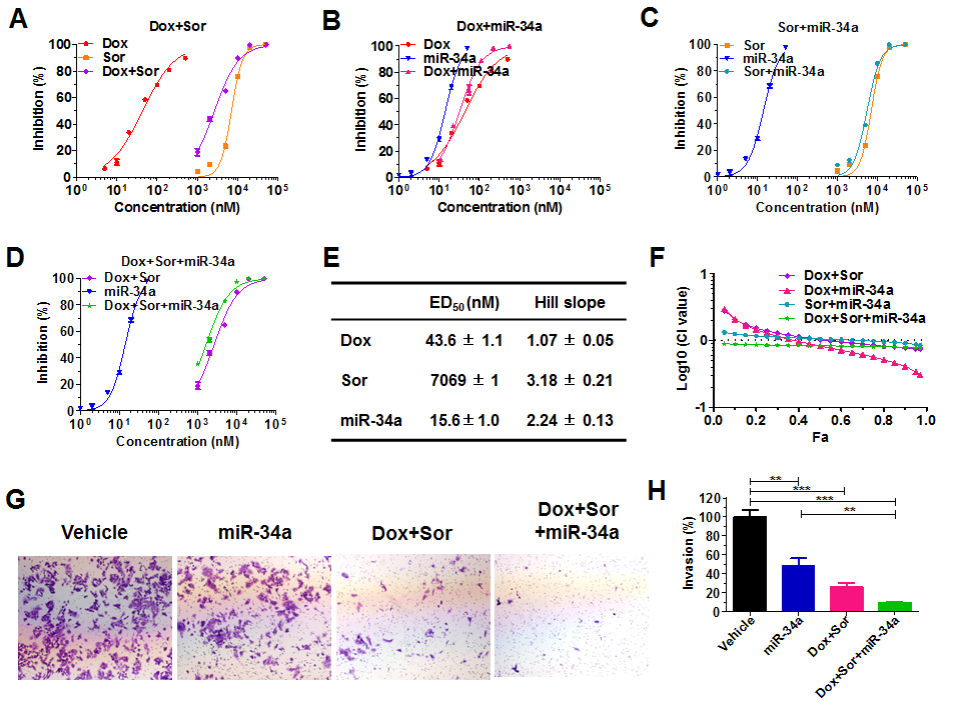
Doxorubicin, sorafenib and miR-34a produced synergism and the greatest effects in suppressing cancer cell proliferation and invasion Dose-response curves for Dox and Sor **A**., Dox and miR-34a **B**., Sor and miR-34a **C**., and Dox plus Sor and miR-34a **D**., alone or in combination (Dox: Sor: miR-34a = 10:1000:1; thus the X-axis indicates the combined drug concentrations), in the inhibition of human osteosarcoma 143B cell proliferation. Cells were treated with various concentrations of Dox, Sor, and miR-34a alone or in combination. Cell viability was determined using CellTiter-Glo luminescent cell viability assay at 72 h post-treatment. **E**. Estimated pharmacodynamic parameters for Dox, Sor and miR-34a in the suppression of 143B cell proliferation. Values are mean ± SD of quadruplicate treatments. **F**. Chou-Talalay (Fa-CI) plot demonstrated the synergistic effects for Dox, Sor and miR-34a in the suppression of 143B cell proliferation. **G**. and **H**. The invasion capability of 143B cells was inhibited to the greatest extent by the triple-drug treatment. 72 h post-treatment, cells were subjected to Matrigel invasion assay for 30 h, fixed with 10% formalin, and then stained with 0.1% crystal violet. Images (G; 40 ×) were acquired with an Olympus IX2-UCB microscope, and invasion capabilities **H**. were compared for different treatments. Values are mean ± SD of triplicate treatments. **P < 0.01, and ***P < 0.001 (one-way ANOVA with Bonferroni post tests).

To define the combinatorial effects of doxorubicin, sorafenib and miR-34a in the inhibition of 143B cell growth, we employed CellTiter-Glo Luminescent Cell Viability Assay to quantitate the anti-proliferative activities of individual drugs and various combinations. As expected, doxorubicin, sorafenib and miR-34a prodrug alone reduced the proliferation of 143B cells in a dose-dependent manner, and a dual- or triple-drug combination was more effective than individual agents (Figure 2A-2F). Interestingly, the anti-proliferation effects of sorafenib and miR-34a combination were mainly additive or even antagonistic at lower concentrations. In contrast, co-administration of doxorubicin with sorafenib or miR-34a or sorafenib plus miR-34a produced synergistic effects in suppressing the proliferation of 143B cells, where the synergism of triple-drug combination persisted at all concentrations (Figure 2F). Therefore, combination therapy could offer remarkable degrees of dose reduction, especially for doxorubicin and miR-34a prodrug, to achieve the same levels of anti-proliferation effects (Supplementary Figure S1A-D). For instance, an 80% inhibition requires approximately 25 nM miR-34a alone or 160 nM doxorubicin alone or 10,000 nM sorafenib alone, whereas only 4 nM miR-34a plus 40 nM doxorubicin and 4,000 nM sorafenib in the triple-drug combination (Figure 2A-2D). Moreover, there was a stronger synergism and much greater extent of dose reduction for doxorubicin at higher degrees of cell inhibition (e.g., > 75%) when used with miR-34a or sorafenib or both, which would ultimately lessen or eradicate the risk of toxicity that might be induced by high doses of doxorubicin.

### Triple-drug combination with doxorubicin, sorafenib and miR-34a almost completely blocks the invasion potential of human 143B cells

We further performed *in vitro* cell invasion assays to determine the impact of triple-drug combination on invasion potential of 143B cells (Figure 2G-2H) since invasion abilities of cancer cells are critical for the local destruction and metastasis of tumors. Our results showed that miR-34a alone suppressed the invasion capability of 143B cells by about 50%, and doxorubicin plus sorafenib reduced cell invasion by 74%, as compared to the vehicle control. Furthermore, triple-drug combination with doxorubicin, sorafenib and miR-34a led to over 90% suppression of cell invasiveness, a significantly greater degree than miR-34a prodrug or doxorubicin plus sorafenib (Figure 2H). The results demonstrate that triple-drug combination was the most effective to block the invasion ability of highly metastatic 143B cells.

### Triple-drug therapy suppresses orthotopic osteosarcoma xenograft tumor progression and spontaneous pulmonary metastases to the greatest levels in mouse models

**Figure 3:**
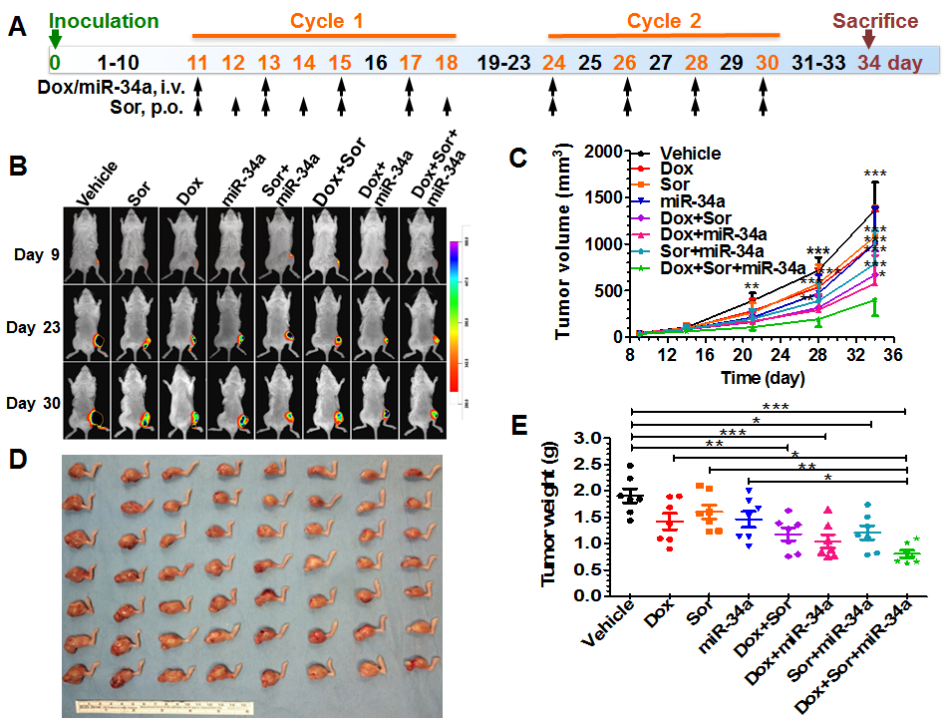
Triple-drug therapy was the most effective in suppressing tumor growth in orthotopic, metastatic osteosarcoma xenograft mouse models **A**. Scheme of therapy study in mouse models including osteosarcoma cell inoculation and drug treatment. **B**. Comparison of bioluminescent signals of orthotopic xenograft tumors on day 9, 23, and 30 among different treatment groups. **C**. Xenograft tumor growth was reduced to a significantly greater degree by triple therapy than vehicle or Dox, Sor, mir-34a alone, or Dox plus Sor, or Sor plus miR-34a (*P < 0.05, **P < 0.01, and ***P < 0.001 as compared to triple treatment; two-way ANOVA with Bonferroni post tests). **D**. Visual comparison of dissected orthotopic osteosarcoma tumor tissues from mice with different treatments. **E**. Weights of the dissected xenograft tumors (*P < 0.05, **P < 0.01, and ***P < 0.001; one-way ANOVA with Bonferroni post tests). Values are mean ± SD (N = 7 per group).

**Figure 4:**
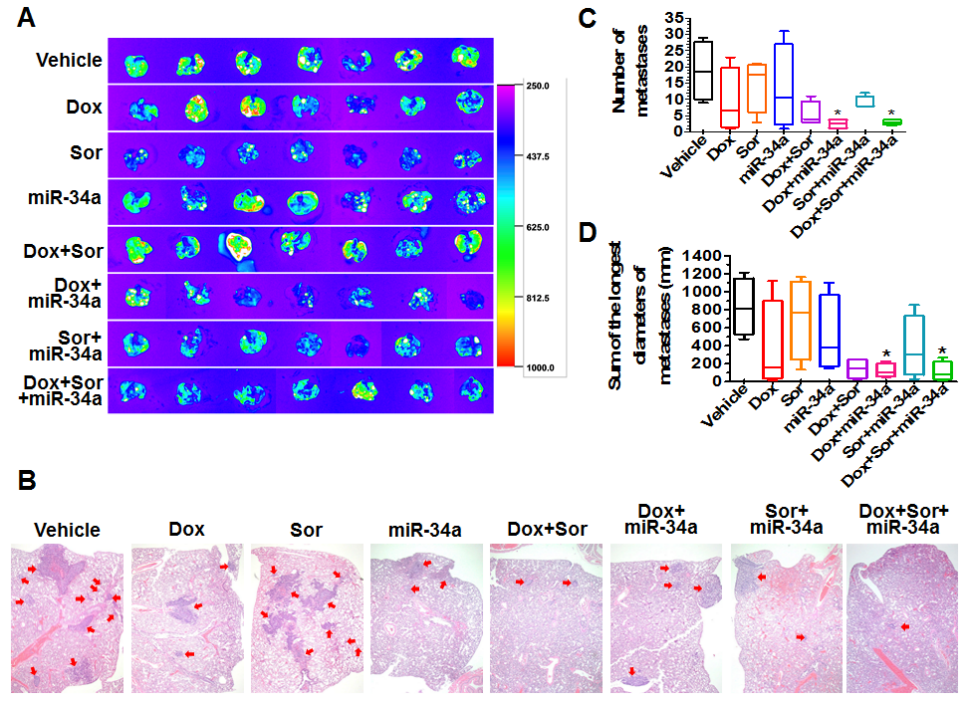
Doxorubicin, sorafenib and miR-34a triple-drug therapy sharply reduced pulmonary metastases from orthotopic osteosarcomas in mouse models **A**. Comparison of *ex vivo* GFP images of lung metastases between different treatment groups (N = 7 per group). **B**. H&E histology (40 ×) showed a much lower degree of tumor metastases (red arrow) for lung tissues dissected from mice under triple drug combination therapy. **C**. and **D**. Comparison of the numbers of lung metastases and sum of the longest diameters of lung metastases, respectively, between different treatments. The boxes extend from the 10th to the 90th percentile, the lines indicate the median values, and the whiskers indicate the range of values. *P < 0.05 as compared to vehicle control group (N = 4 per group).

We thus established an orthotopic osteosarcoma/spontaneous metastases mouse model to determine the effectiveness of rationally-designed triple-drug therapy in the suppression of xenograft tumor growth (Figure 3) and reduction of spontaneous pulmonary metastases (Figure 4). The sizes of orthotopic xenograft osteosarcomas were monitored over time through bioluminescence imaging and by caliper. Our data showed that every drug treatment could significantly inhibit the outgrowth of viable xenograft osteosarcomas than vehicle control, albeit to different degrees, as manifested by bioluminescence intensities (Figure 3B) and calculated tumor volumes (Figure 3C). The degrees of suppression by triple-drug therapy were significantly greater than sorafenib plus either doxorubicin or miR-34a treatment (Figure 3C). Although statistical significance was not reached when triple-drug therapy group was compared to doxorubicin plus miR-34a treatment, the greatest extent of repression of osteosarcoma progression by triple therapy was obvious (Figure 3C). The consistent tendency was also indicated by visual inspection of dissected xenograft osteosarcomas (Figure 3D) and quantitative measurement (Figure 3E) of tumor weights.

To evaluate the effects of individual treatments on pulmonary metastases, lung tissues were dissected from all mice and GFP-expressing tumors were imaged. As demonstrated by *ex vivo* GFP signals including the distributions and intensities (Figure 4A), triple-drug combination therapy showed a much greater suppression of pulmonary metastases. We thus randomly selected four samples from each group for histopathological analyses, among which triple therapy group consistently showed the least lung metastases (Figure 4B). Specifically, the numbers of metastases per lung sample were much less in triple-drug therapy groups (1-4 foci of metastases in each lung) than vehicle control group (9-34 foci) (Figure 4C). In addition, the sum of the longest diameters of metastasis foci in triple-drug therapy group as well as doxorubicin/miR-34a treatment group was significantly smaller than vehicle control (Figure 4D). Together, the results demonstrated that triple-drug therapy using doxorubicin, miR-34a and sorafenib provided the greatest degrees of protection against orthotopic osteosarcoma progression and pulmonary metastases.

### Triple-drug combination therapy is well tolerated in tumor-bearing mice

**Figure 5:**
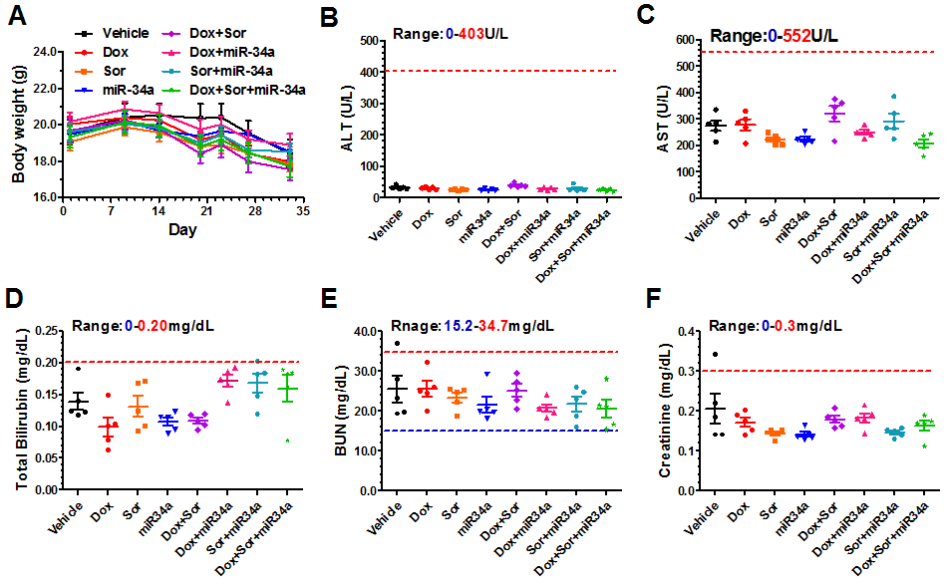
Triple-drug combination therapy was well tolerated in mice Compared to vehicle control group, systemic (co-)administration of doxorubicin, sorafenib and bioengineered miR-34a prodrug had no significant impact on mouse body weights **A**. and blood chemistry profiles including alanine transaminase (ALT; **B**.), aspartate transaminase (AST; **C**.), total bilirubin **D**., blood urea nitrogen (BUN; **E**.), and creatinine **F**. levels. Values are mean ± SD (N = 5 in each group). The ranges of individual blood chemistry biomarkers (derived from BALB/c mice; Comparative Pathology Laboratory at UC-Davis) were marked as references. In addition, cTnI levels in all mice were below the lower limit of quantification (0.156 ng/ml), indicating the absence of cardiac toxicity.

Following the first course of treatment (Figure 3A) mouse body weights decreased to different degrees, which was more obvious in doxorubicin-treated groups (Figure 5A) and might indicate some doxorubicin-associated toxicity. However, all mice did not show any signs of stress such as hunched posture or labored movement. After five-day recovery showing gain of body weights (Figure 5A), the second course of treatment was initiated with slightly reduced drug doses to avoid any potential adverse effects. After this treatment, all mice including those treated with vehicle showed 5-10% loss of body weights (Figure 5A), likely due to the progression of osteosarcoma (Figure 3), severe metastases burden (Figure 4) and/or handling rather than drug treatment. There was actually one mouse in the vehicle treatment group that showed the greatest degree of body weight loss at the last week of study, which was mainly attributable to cancer-associated cachexia resulting from advanced osteosarcoma.

To examine the risk of hepatic and renal toxicities, serum samples were collected and subjected to blood chemistry profiling (Figure 5B-5F). Levels of alanine aminotransferase (ALT), aspartate aminotransferase (AST), total bilirubin, both blood urea nitrogen (BUN) and creatinine concentrations were all variable in individual treatment groups but within the normal ranges (except the cachectic mouse described above), the latter of which indicates the absence of liver and kidney toxicity. In addition, serum cardiac troponin I (cTnI) levels [33] were examined using an ELISA assay for the assessment of risk of doxorubicin-associated myocardial damage. Our data showed that cTnI levels in control and treatment groups were all under the lower limit of quantification (0.156 ng/ml), suggesting the lack of cardiac toxicity. Together, these results indicated that therapeutic doses of doxorubicin, sorafenib and miR-34a prodrug alone or in combination were well tolerated in these mice.

### Triple-drug combination therapy improves the overall survival to the greatest degree in experimental metastases mouse models

**Figure 6:**
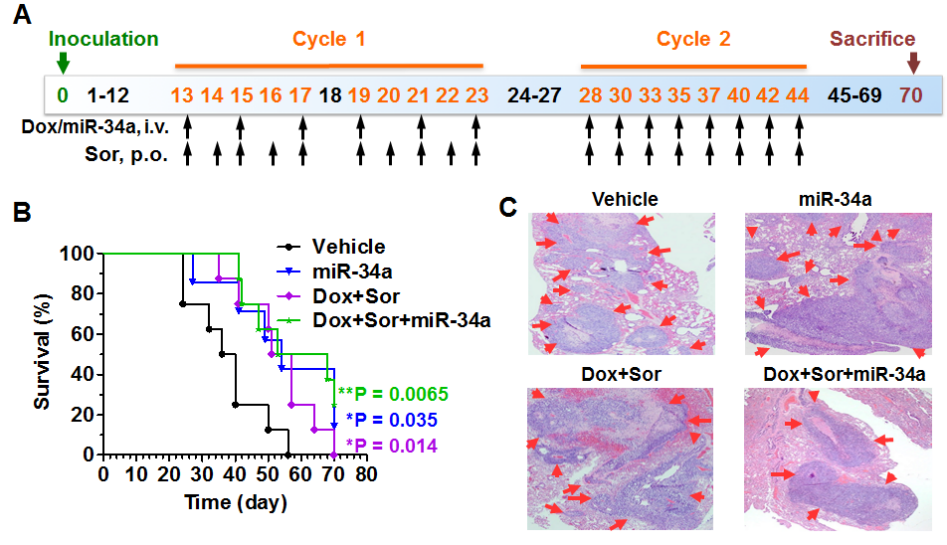
Triple-drug therapy improves overall survival to the greatest level in experimental lung metastases mice **A**. Timeline of osteosarcoma cell inoculation to produce artificial pulmonary metastases mouse models, and drug treatment. **B**. Survival analysis showed that mice in treatment groups lived much longer than vehicle control (*P < 0.05, **P < 0.01; N = 7-8 per group; Log-rank (Mantel-Cox) Test). Median survival was 38 days for mice treated with vehicle, 54 days for miR-34a alone, 54 days for doxorubicin (Dox) plus sorafenib (Sor), and 60.5 days for doxorubicin, sorafenib plus miR-34a triple-drug therapy. **C**. H&E histology (40 ×) confirmed that mouse lungs were severely eroded by the tumor metastases (red arrow).

To further define the benefits of triple therapy for the treatment of late-stage metastases, we established an artificial pulmonary metastases mouse model *via* intravenous injection of 143B OS cells (Figure 6A). Metastases were confirmed to be visible on the surface of all mouse lung tissues at about 2 weeks post-inoculation in our pilot study. Therefore, we started the treatment with vehicle, miR-34a, doxorubicin/sorafenib, and the triple-drug combination on day 13 post-inoculation (Figure 6A). The survival study was terminated on day 70 when all mice treated with vehicle or doxorubicin/sorafenib died (Figure 6B). The presence of extensive pulmonary metastases after death or euthanasia was verified by necropsy and histological analyses (Figure 6C). Furthermore, extrapulmonary metastases (e.g., skull, jaw, shoulder, knee or/and caudal vertebra) were noted in 3 mice among each group treated with vehicle, miR-34a or doxorubicin plus sorafenib, in contrast to 2 mice treated with triple-drug combination.

While doxorubicin plus sorafenib therapy or miR-34a alone significantly increased the overall survival and improved the median survival from 38 days to 54 days, triple-drug combination therapy improved the overall survival to a more significant level and extended the median survival to an even longer time (60.5 days; Figure 6B). In contrast to the death of all mice treated with vehicle control by day 56, 50% of the mice under triple-drug therapy were still alive on day 56 and 25% of mice were alive at the termination of the study (Figure 6B) even though palpable pulmonary metastases were identified (Figure 6C). These results demonstrated that triple-drug therapy with DNA-intercalating doxorubicin, RNA-interfering miR-34a and protein kinase-inhibiting sorafenib could ultimately extend the survival.

## DISCUSSION

Treating lethal metastasis might not be feasible through the inhibition of single target or pathway due to the presence of multiple and/or complementary mechanisms underlying complex cancer invasion-metastasis cascade [3]. In contrast, combination therapy has been proved as an effective means to fight against metastatic cancers [12]. Nevertheless, present combination therapies all focus on the co-inhibition of protein targets. For instance, the first immune checkpoint inhibitor combination approved by FDA in October, 2015 for the treatment of unresectable or metastatic BRAF^V600^ wild-type melanoma consists of nivolumab and ipilimumab, which target the programmed cell death protein 1 and cytotoxic T lymphocyte antigen 4, respectively [34]. In this study, we demonstrated a new strategy to combat metastasis, which utilizes multiple drugs to co-target DNA, mRNA and protein molecules critical for many cellular processes. The benefits of “saturation attack” were strikingly obvious, given the greatest degrees in the suppression of orthotopic osteosarcoma progression and lung metastases, as well as improvement of survival in spontaneous and experimental metastases mouse models, which were attributable to the independent and complementary or combined actions of model drugs on DNA replication, mRNA translation, and protein signal transduction in cells.

Doxorubicin, biological miR-34 prodrug, and sorafenib were chosen as model drugs to target DNA, RNA and protein molecules, respectively. The mechanistic actions of individual model drugs on DNA replication, target mRNA translation, and protein signal transduction were illustrated by an increased γH2A.X foci formation, reduced c-MET expression, and decreased Erk1/2 phosphorylation, respectively, in human OS 143B cells, which carry a *p53* mutant and show high levels of p53 protein expression commonly identified in human malignant OS, thus representing a clinically-relevant OS cell model [35, 36]. While sorafenib or/and miR-34a alone had no or minimal effects on γH2A.X foci formation, both could largely enhance doxorubicin-provoked DNA damage in 143B cells. By contrast, the reduction of pErk1/2 levels by sorafenib alone seemed to reach the maximal effects as the addition of doxorubicin and miR-34a did not show any impact. Consistent with the upregulation of c-MET in ovarian and hepatocellular carcinoma cells by doxorubicin and sorafenib that may be linked to drug resistance [37, 38], our data revealed higher levels of c-MET in OS cells treated with doxorubicin and sorafenib, alone or in combination. However, the inclusion of c-MET mRNA-targeting miR-34a completely erased such negative impact of doxorubicin and sorafenib, highlighting the benefits of independent and complementary actions of rationally-combined DNA-intercalating, RNA-interfering, and protein-inhibiting drugs.

The strong synergism of doxorubicin, sorafenib and miR-34a allowed high degrees of dose reduction of individual drugs to achieve the same level of efficacy (e.g., Fa = 0.8). This is of particular important for chemotherapeutic doxorubicin and RNAi drug miR-34a, which at higher doses might induce cardiotoxicity and immune responses, respectively. As a result, relatively lower doses of individual drugs, alone or in combination, were utilized to evaluate saturation attack strategy in the mouse model of pulmonary metastases from orthotopic OS xenografts, which resembles the clinical features of human OS for the evaluation of new therapeutic strategies [3, 39]. Besides the body weight loss caused by severe tumor burden (vehicle control), drug-associated body weight loss was noted but acceptable in all treatment groups. Furthermore, the drug-/injection-induced body weight was restorable after withdrawal. All mice showed normal blood chemistry profiles, except for one mouse in the vehicle control group with cancer-related cachexia and abnormal blood BUN and creatinine levels, indicating that therapeutic doses of doxorubicin, sorafenib and bioengineered miR-34a prodrug were well tolerated in tumor-bearing mice without causing any liver or kidney toxicity. In addition, no mice showed any cardiac injury, as determined by the levels of cTnIs. This may be due to the use of much lower doses of doxorubicin in the present study, although it is unknown if co-administration of miR-34a prodrug would give rise to asymptomatic heart dysfunction of doxorubicin plus sorafenib [40].

The benefits of triple-drug therapy *via* co-targeting DNA, RNA and protein molecules were notably demonstrated by the greatest level of efficacy in the spontaneous metastases mouse models. Compared to vehicle control, single drug treatment offered a lower degree of suppression of orthotopic OS xenograft tumor growth and showed insignificant effects on pulmonary metastases. In addition to consistent findings on the effectiveness of doxorubicin chemotherapy plus miR-34a replacement therapy on OS xenograft tumor growth [17], present study revealed much greater degrees of suppression by the combination of doxorubicin plus sorafenib, and sorafenib plus miR-34a than single drug treatment. Furthermore, co-administration of doxorubicin and miR-34a significantly suppressed lung metastases in these mice although the combinations of sorafenib plus miR-34a and doxorubicin plus sorafenib only had modest effects. Most importantly, the greatest degree of suppression of orthotopic OS xenograft tumor growth and pulmonary metastases were identified in mice treated with triple-drug combination. The optimal therapeutic outcomes from saturation attack observed *in vivo* are also consistent with the synergistic effects in the control of cell proliferation and the blockage of invasiveness identified *in vitro*.

The present study further revealed the benefit of triple-drug therapy in experimental metastasis mouse model, as indicated by the extents of improvement of survival. Consistent with the encouraging results of doxorubicin plus sorafenib therapy for advanced HCC patients [40], dual-drug combination was able to significantly improve the survival of pulmonary metastatic mice. Furthermore, two cycles of monotherapy with bioengineered miR-34a prodrug significantly improved the survival rate of mice. This is in agreement with sufficient distribution of *in vivo*-jetPEI-encapsulated miR-34a prodrug to mouse lung tissues (unpublished data) and the actions of miR-34a on multiple therapeutic targets including PD-L1 implicated in immunotherapy [41, 42]. The triple-drug combination therapy enhanced mouse survival rate to the most significant level, which was also indicated by the longest median survival. The absence of statistical differences between triple-drug combination therapy and miR-34a alone or doxorubicin/sorafenib is likely due to a quite heavy burden of lung metastases in the experimental animal models and relatively small sample size in each group.

In summary, we have demonstrated that a rational triple-drug combination therapy can offer robust and optimal outcomes in spontaneous and experimental metastases mouse models, as manifested by the greatest degrees of suppression of tumor progression and metastasis as well as extension of overall survival. Independent and combined actions of model drugs doxorubicin, miR-34a and sorafenib on the inhibition of DNA replication, RNA translation and protein signal transduction are indicated by the increase of γH2A.X, decrease of c-MET expression and reduction of ERK1/2 phosphorylation, respectively. Although the identification of optimal dose regimens for direct clinical application will be challenging, rational combination therapy offers obvious synergism and permits the use of much lower and safe doses of drugs. Our findings suggest that co-targeting critical DNA, RNA and protein molecules may provide a framework for developing new strategies for the treatment of the deadliest metastases.

## MATERIALS AND METHODS

### Materials

Doxorubicin (hydrochloride salt, > 99%) and sorafenib (p-toluenesulfonate salt, > 99%) were purchased from LC Laboratories (Woburn, MA, USA). RPMI 1640 medium, trypsin and phosphate-buffered saline (PBS), fetal bovine serum, opti-MEM, BCA Protein Assay Kit, and Lipofectamine 3000 Transfection Kit were bought from Thermo-Fisher Scientific Inc (Waltham, MA, USA). RIPA buffer was bought from Sigma-Aldrich (St. Louis, MO, USA) and protease inhibitor cocktail was from Roche Diagnostics (Mannheim, Germany). Bovine Serum Albumin and *in vivo*-jetPEI were purchased from VWR (Visalia, CA, USA). ECL substrate and PVDF membrane were supplied by Bio-Rad (Hercules, CA, USA). Crystal violet was bought from MP Biomedicals, LLC (Solon, OH, USA). Manufacturers of antibodies used for western blot and immunofluorescence were shown in Supplementary Table S1. All other chemicals and organic solvents of analytical grade were purchased from Sigma-Aldrich or Thermo-Fisher Scientific Inc.

### Biologic miR-34a prodrug

Bioengineered miR-34a prodrug (tRNA/pre-miR-34a) was expressed in *E. coli* (strain HST08) and purified by an anion exchange fast protein liquid chromatograph (FPLC) method, as described [13, 14, 43]. The purity of miR-34a prodrug was assessed by denaturing urea polyacrylamide gel electrophoresis (PAGE) and determined quantitatively by high performance liquid chromatography (HPLC) analysis [14]. Endotoxin activities were determined using the Pyrogent-5000 kinetic LAL assay (Lonza) by following the instructions. The miR-34a prodrugs over 98% pure and less than 10 EU/μg RNA were used in the following study. Lipofectamine 3000 and *in vivo*-jetPEI were used for the delivery of miR-34a to cells and animals, respectively.

### Human cell lines

The human OS cell lines 143B (CRL-8303) was bought from American Type Culture Collection (Manassas, VA, USA) and cultured in RPMI 1640 medium containing 10% FBS. The luciferase and GFP-expressing 143B-GFP-Luc cells were generated after transduction with pCCLc-Luc-EGFP lentiviral constructs (Vector Core, UC Davis Medical center, Sacramento CA).

### Cell viability assay, dose-response relationship and synergism

143B-GFP-Luc cells (5 × 10^3^ cells/well) were seeded in 96-well plate overnight and then treated with different concentrations of doxorubicin (0, 5, 10, 20, 50, 100, 200, 500 nM), sorafenib (0, 1, 2, 5, 10, 20, 50 μM), miR-34a prodrug (0, 1, 2, 5, 10, 20, 50 nM) or the combination in fixed ratio (doxorubicin: sorafenib: miR-34a, 10:1000:1). After 72 hours, cell viability was determined by CellTiter-Glo Luminescent Cell Viability Assay (Promega, Madison, WI, USA). The degrees of inhibition were calculated by adjusting the vehicle control group to 0% and dose-response curves were established by plotting inhibition *versus* drug concentration. Cells were treated in quadruplicate and assayed separately. Data were fit to a normalized inhibitory dose-response model with variable slope (Y = 100/(1+10^((LogEC50-X)*HillSlope); GraphPad Prism, San Diego, CA) for the estimation of EC50, Hill slope, and Bottom values.

The Chou-Talalay approach [44] was employed to further analyze interactions of these three drugs in the inhibition of human OS 143B-GFP-Luc cell growth. For triple therapy, doxorubicin and sorafenib were used as an entirety to combine with miR-34a. The combination index (CI) values at each fraction affected (Fa) were calculated with CompuSyn software (ComboSyn, Inc., USA) to define the combinational effects, i.e., CI < 1 indicates synergism, CI = 1 indicates additivity, and CI > 1 indicates antagonism. In addition, the dose reduction index (DRI) values were calculated for the evaluation of dose reduction levels in combination therapy [44].

### Invasion assay

Invasive potential of 143B-GFP-Luc cells, treated with vehicle, or 12 nM miR-34a, or 40 nM doxorubicin plus 4 μM sorafenib, or the combination for 72 h, was assessed as described previously [16, 17, 45] by using Corning BioCoat Matrigel Invasion Chamber with 8.0 mm PET membrane coated with matrigel (Corning, NY, USA). Briefly, 5 × 10^4^ cells in 500 μl serum-free RPMI 1640 media were placed into the upper insert, and 750 μl medium supplemented with 10% FBS was added into the well as a chemo-attractant. 30 h later, cells in the upper inserts were removed and the lower were fixed with 10% formalin, stained with 0.1% crystal violet, and then photographed with five fields per insert under an Olympus IX2-UCB microscope (40 × magnifications). Each treatment group was conducted in triplicate and assessed independently. The quantification of invaded cells was calculated using Image J software and invasion capacity was determined by adjusting the vehicle control group to 100%.

### Immunofluorescence study on γH2A.X foci

143B-GFP-Luc Cells (1 × 10^5^ per well) were plated into 6-well plates with coverslips on the bottom and then treated with vehicle, doxorubicin (40 nM), sorafenib (4 μM) and miR-34a (12 nM) alone or in combination on the next day. 24 hours later, cells were fixed with 10% formaldehyde for 15 min at room temperature, rinsed three times in PBS, and blocked for 1 h in PBS consisting of 2% bovine serum albumin/0.3% Triton X-100 (Fisher Scientific) at room temperature. After rinsed three times in PBS, cells were incubated with anti-γH2A.X (Ser139) antibody (1:800, Cell Signaling Technology, Danvers, MA) overnight at 4 °C. After washed with PBS for 3 times, cells were incubated in Alexa Fluor^®^ 488-conjugated goat anti-rabbit IgG (1:800, Cell Signaling Technology) for 1 h at room temperature in dark. Nucleus was counterstained with DAPI using Prolong^®^ Gold Antifade Reagent (Cell Signaling Technology). Images were taken with a Zeiss Axio Observer.z1 Microscope coupled to a Zeiss LSM 710 Scanning Device (Zeiss, Oberkochen, Germany).

### Protein isolation and immunoblot analysis

Human OS 143B-GFP-Luc cells were treated with doxorubicin (40 nM), sorafenib (4 μM) and miR-34a (12 nM) alone or in combination. Cells were collected at 72 h post-treatment and lysed in RIPA buffer containing protease inhibitor cocktail. Following determination of protein concentrations with a BCA Protein Assay Kit, protein samples (30 μg/lane) were loaded and separated on a 10% SDS-PAGE gel and transferred onto PVDF membranes. Membranes were incubated with anti-c-MET (1:200; C-28; Santa Cruz, Dallas, Texas), anti-p-Erk1/2 (1:1000; Thr202/Tyr204; Cell Signaling Technology), or anti-GAPDH (1:2,000; FL-335; Santa Cruz) rabbit antibody, and then with a peroxidase goat anti-rabbit IgG (Jackson ImmunoResearch Inc., West Grove, PA, USA). After incubation with Clarity Western ECL substrates (Bio-Rad, Hercules, CA, USA), the proteins were visualized with the ChemiDoc MP Imaging System (Bio-Rad) and quantified by Image Lab software (Bio-Rad). GAPDH was used as a loading control.

### Animal models and therapy studies

All animal procedures were approved by the Institutional Animal Care and Use Committee of University of California, Davis, and all animal studies were conducted in accordance with the relevant national and international guidelines. Non-obese diabetic/ severe combined immunodeficient (NOD/SCID) mice (NOD.CB17-Prkdcscid/J) were purchased from Jackson Laboratory and adaptively fed at least one week before experiments. Animals were randomly assigned to treatment groups and closely monitored. Mice were euthanized if tumor size reached 2,000 mm^3^ (roughly 10% baseline body weight) or exceeded 2 cm in any direction, or body weight decreased 20%, otherwise, they were euthanized at the end of the study.

To establish orthotopic osteosarcoma xenograft/spontaneous metastasis mouse models, 143B-GFP-Luc cells (7.2 × 10^5^ cells suspended in 40 μl PBS) were injected into the right tibia of anesthetized 7-week-old female mice [36, 46]. Tumor growth was monitored by bioluminescence imaging and measured with a caliper. In particular, tumor volume was calculated using the formula, tumor volume (mm^3^) = (length + width) × length × width × 0.2618 [16, 36]. For bioluminescent imaging, D-luciferin (150 mg/kg) (BioVision, Inc. Milpitas, CA, USA) was injected into the abdominal cavity of anesthetized mice immediately before imaging. Images were acquired by KODAK Image Station 4000 Digital Imaging Systems and analyzed by Carestream Molecular Imaging Software 5.4.2 (Carestream Health, Inc.). On day 11 after inoculation, 56 mice were divided into 8 groups (7 mice in each group) according to the tumor volume and bioluminescent intensity to receive two cycles of treatments. The 1^st^ cycle consisted of doxorubicin (12 μg/mouse, intravenously (IV), every other day (QOD) for 4 times), sorafenib (200 μg/mouse, intragastrically (IG), every day (QD) for 7 times), or/and miR-34a (12 μg/mouse, IV, QOD for 4 times). The 2^nd^ cycle consisted of doxorubicin (10 μg/mouse, IV, QOD for 4 times), sorafenib (200 μg/mouse, IG, QOD for 4 times) or/and miR-34a (10 μg/mouse, IV, QOD for 4 times). At the end of the study, all mice were sacrificed and tumors were dissected and weighed. The lung tissues were dissected for *ex vivo* GFP imaging and fixed in 10% formalin, and then subjected to hematoxylin and eosin (H&E) staining for histological evaluation in the Clinical Immunohistochemistry Laboratory at Roswell Park Cancer Institute (Buffalo, NY, USA). Additionally, serum was isolated using a serum separator (BD Biosciences, San Jose, CA, USA) and then subjected to blood chemistry profiling in the Comparative Pathology Laboratory at UC Davis. Serum cardiac troponin-I (cTnI) was analyzed with a Mouse Cardiac Troponin-I ELISA Kit (Life diagnostics, Inc. West Chester, PA, USA).

To establish artificial metastasis mouse models for survival study, 9-week-old female mice were inoculated with 5 × 10^6^ 143B-GFP-Luc cells in 250 μl PBS per mouse *via* the tail vein injection. 12 days later, the mice were randomized to 4 groups to receive two cycles of miR-34a, doxorubicin/sorafenib, doxorubicin/sorafenib+miR-34a or control vehicle treatment. The 1^st^ cycle consisted of doxorubicin (12 μg/mouse, IV, QOD for 6 times), sorafenib (200 μg/mouse, IG, QD for 10 times), or/and miR-34a (12 μg/mouse, IV, QOD for 6 times). The 2^nd^ cycle consisted of doxorubicin (10 μg/mouse, IV, QOD for 8 times), sorafenib (200 μg/mouse, IG, QOD for 8 times) or/and miR-34a (10 μg/mouse, IV, QOD for 8 times). Body weight loss over 10% was considered as death. All mice underwent complete necropsy for the confirmation of experimental pulmonary metastases. Survival was analyzed by Kaplan-Meier method and compared by Log-rank (Mantel-Cox) Test (GraphPad Prism).

### Statistics analysis

Statistical analysis was performed using GraphPad Prism. Depending upon the groups and variances, data were analyzed with one-way or two-way ANOVA. Additionally, Bonferroni post-tests were applied for multiple comparisons. Difference was considered as statistically significant at the level of *P < 0.05*.

## SUPPLEMENTARY MATERIALS FIGURE AND TABLE


