# Supplementary material for: Co-targeting of DNA, RNA, and protein molecules provides optimal outcomes for treating osteosarcoma and pulmonary metastasis in spontaneous and experimental metastasis mouse models
Source: Oncotarget. 2017 Mar 18;8(19):30742–55. doi: 10.18632/oncotarget.16372 (PMC5458164; doi:10.18632/oncotarget.16372)
Supplement: Supplementary file 1 [file oncotarget-08-30742-s001.pdf]

Co-targeting of DNA, RNA, and protein molecules provides optimal outcomes for treating osteosarcoma and pulmonary metastasis in spontaneous and experimental metastasis mouse models

Supplementary Material

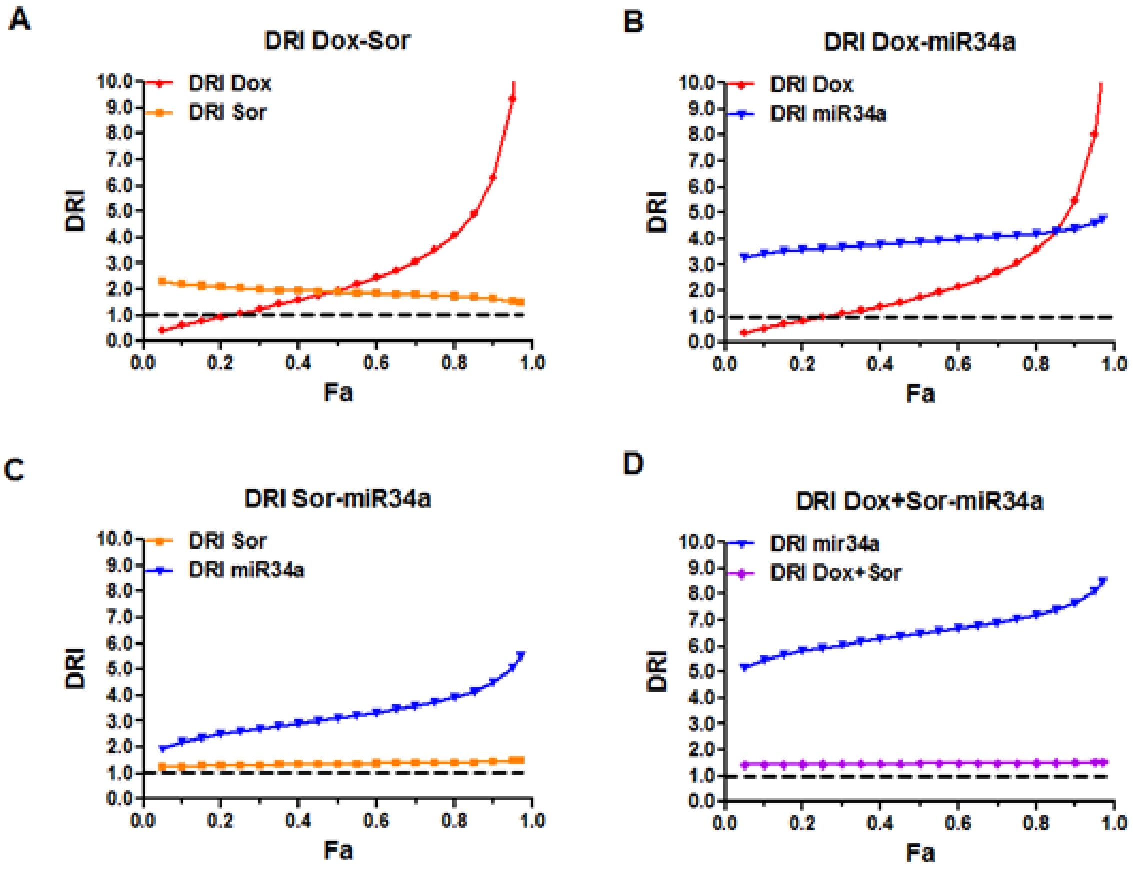

Supplementary Figure S1: Fa-DRI plots show the levels of dose reduction of individual drugs when used in combination. Among them, the doses of doxorubicin and miR-34a prodrug could be reduced to much greater degrees.

Supplementary Table S1: Antibodies used for Western blots and immunofluorescence analyses.

**Antibodies**	**Manufacturer**	**Cat No.**
γH2A.X	Cell Signaling	2577
C-MET	Santa Cruz	sc-514148
Phopsho-Erk1/2	Cell Signaling	9101
GAPDH	Santa Cruz	sc-25778
Peroxidase-conjugated goat anti-rabbit IgG	Jackson ImmunoReseach	111-035-003
Alexa Fluor® 488-conjugated goat anti-rabbit IgG	Cell signaling	4412
